# Large-scale replication study identified multiple independent SNPs in *RET* synergistically associated with Hirschsprung disease in Southern Chinese population

**DOI:** 10.18632/aging.101294

**Published:** 2017-09-20

**Authors:** Yan Zhang, Qiuming He, Ruizhong Zhang, Hong Zhang, Wei Zhong, Huimin Xia

**Affiliations:** ^1^ Department of Pediatric Surgery, Guangzhou Institute of Pediatrics, Guangzhou Women and Children's Medical Center, Guangzhou Medical University, Guangdong, China

**Keywords:** Hirschsprung disease, epistasis, association, subclinical stratification

## Abstract

Hischsprung disease (HSCR) is an intestinal disorder with strong genetic components. *RET* was considered as the strongest contributor. Multiple single nucleotide polymorphisms (SNP) were demonstrated as associated with HSCR in different populations. However, whether the associations of reported SNPs derived from one causal variants or congregations of multiple variants were still not clear. In this study, we successfully genotyped 16 SNPs in *RET* with a largest case-control study to date, totaling 1470 HSCR and 1473 control subjects in South Chinese population. Multiple independent contributors were identified through pairwise and stepwise logistic regression. The intragenic synergistic effect among these SNPs were further explored and cross validated by logistic regression and multifactor dimensionality reduction (MDR). Noteworthy, in further subclinical manifestation analysis, the six potential independent contributors in *RET* were more essential for the patients with short-segment aganglionosis (S-HSCR). Although functional evaluations are required, our comprehensive analysis for *RET* gene integrating detailed disease subphenotypes might facilitate improved understanding for the genetic understanding of HSCR etiology.

## INTRODUCTION

Hirschsprung disease (HSCR) is an intestinal disorder characterized by the absence of nerves in parts of the intestine. HSCR occurs in approximately 1 in 5,000 newborns [1]. Hirschsprung disease affects all races; however, it is roughly 3 times more common among Asians. This disease occurs more often in males than in females, with a male-to-female ratio of approximately 4:1 [2]. Hirschsprung disease can be defined in to 3 types by the length of intestine lacking nerve cells, known as short-segment HSCR (S-HSCR), long-segment HSCR (L-HSCR) and total colonic aganglionosis (TCA) [3].

HSCR has a complex genetic etiology. Isolated Hirschsprung disease can result from mutations in one of several genes, including the *RET*, *EDNRB*, and *EDN3* genes [4]. More than 100 *RET* mutations are known in familial and syndromic HSCR patients [5]. However, *RET* mutations have been detected in only up to 50% of familial patients and in 7%-35% sporadic cases [5]. There is growing evidence showing that some potentially functional single nucleotide polymorphisms (SNPs) of *RET* gene could act as susceptibility factors and modify the phenotype of HSCR, especially in certain combinations of alleles, haplotypes [6]. Specifically, Rs2435357 underlies HSCR risk in cases with both European and Asian ancestry [7]. Rs2435357 maps to intron 1 of *RET* and the disease-associated allele disrupts binding of *SOX10*, a key enteric nervous system (ENS) transcription factor [7]. Moreover, several SNPs were subsequently replicated in multiple studies [8-10]. However, whether those SNPs derived from multiple independent signals or come out from one single casual mutation was unclear and seldom investigated. Ke [11] demonstrated the presence of multiple independent effects in loci with disease susceptibility, and illustrated that the variance explained by the multiple effects in a locus was much higher than the variance explained by the single SNP reported. Thus, further exploration of those independent effects will not only enrich the list of HSCR susceptibility genes, but also has a potential to advance our understanding on the etiology of this complicated disease.

The joint gene-gene effects may also have a substantial impact on the risk of Hirschsprung disease. Gunadi and colleagues identified the significant synergistic interactions between *RET* and *NRG1* polymorphisms with risks of HSCR at *NRG1* conditional on rs2435357 genotype [12]. Pontual et al. observed enrichment of *RET* hypomorphic alleles in BBS-HSCR patients might underlie a synthetic interaction at a higher level of systems organization [13]. Other than these studies, as the most important gene identified so far, little is known about whether there exist intragenic epistasis effect in *RET* to boost the gene function for disease suscep-tibility.

In this study, we conducted a replication study of 16 SNPs in *RET* using 1470 HSCR cases and 1473 controls to evaluate the independent contribution among them. Fifteen of them were identified as associated with HSCR. Six of them were illustrated as independent contributors for the disease which may derive from different casual variants. Furthermore, the synergistic interactions of these SNPs were investigated on disease association. A SNP sub-network in *RET* is further con-structed. Our results demonstrated that there were mutual significant interactions among six independent SNPs in this locus elevated risk to HSCR, especially in those with specific subclinical manifestations. The role of these variants in gender manifestation differences and influence on the severity of the disease were also examined.

## RESULTS

### Association of multiple RET SNPs with HSCR

Sixteen SNPs centered by SNP rs2435357 aggregating in a 400kb span were selected for replication in South Chinese population using 1470 cases and 1473 controls, to identify multiple independent variants associated with the disease (The selection criteria were listed in Method). Fifteen SNPs shows significant association with HSCR (6.3E-65≤*P*_adj≤2.8E-03) (Table 1).

**Table 1:** Replication results on sixteen selected SNPs in RET in South Chinese population using 1470 cases and 1473 controls

CHR	SNP	BP	Func.refgene	Gene.refgene	A1/A2	F_A	F_U	P	P_adj	OR
10	rs2506030	42952399	intergenic	*BMS1,LINC01264*	G/A	0.80	0.73	3.2E-09	1.9E-07	1.41(1.24~1.61)
10	rs10900297	43077063	upstream	*RET*	C/A	0.82	0.76	2.0E-03	2.8E-03	1.44(1.13~1.82)
10	rs2506011	43079488	intronic	*RET*	T/C	0.81	0.66	1.1E-39	5.3E-32	2.19(1.92~2.49)
10	rs2435357	43086608	intronic	*RET*	T/C	0.71	0.44	9.5E-94	1.6E-65	2.80(2.49~3.15)
10	rs2435356	43087702	intronic	*RET*	A/G	0.70	0.44	2.6E-88	3.7E-62	2.71(2.41~3.05)
10	rs2505532	43099097	intronic	*RET*	C/T	0.75	0.56	1.2E-50	2.9E-38	2.21(1.96~2.49)
10	rs2565206	43100333	intronic	*RET*	G/T	0.93	0.88	1.1E-13	1.9E-11	1.98(1.63~2.42)
10	rs1800858	43100520	exonic	*RET*	A/G	0.69	0.43	1.2E-85	5.8E-60	2.62(2.34~2.95)
10	rs1800860	43111239	exonic	*RET*	G/A	0.84	0.78	6.3E-09	2.3E-08	1.51(1.30~1.74)
10	rs2742234	43117161	intronic	*RET*	C/T	0.69	0.47	4.7E-61	6.9E-46	2.35(2.09~2.64)
10	rs1800861	43118395	exonic	*RET*	G/T	0.69	0.47	5.2E-64	7.2E-48	2.36(2.10~2.65)
10	rs17158558	43124887	exonic	*RET*	C/T	0.98	0.98	0.09	0.35	1.22(0.81~1.86)
10	rs2742236	43125103	intronic	*RET*	G/A	0.81	0.68	5.8E-30	3.0E-25	2.00(1.76~2.28)
10	rs7893332	43157312	intronic	*CSGALNACT2*	T/G	0.92	0.88	5.5E-07	1.9E-06	1.57(1.30~1.89)
10	rs1254958	43206695	intronic	*RASGEF1A*	T/C	0.69	0.47	1.2E-65	2.6E-48	2.36(2.10~2.65)
10	rs2505526	43274443	intergenic	*RASGEF1A, FXYD4*	G/A	0.66	0.51	1.1E-33	6.8E-28	1.90(1.69~2.13)

CHR: Chromosome; SNP: Single Nucleotide Polymorphism; BP: Base pair of where the SNP is located. Func.refgene: The function role of SNP in the gene. Gene.refgene: The gene where the SNP located to; A1/A2 indicates the risk allele and protective allele to disease; F_A/F_U indicates risk allele frequency of the SNP in cases or controls. The P value indicates the significance based on allelic association tests. P_adj indicates the significance based on the logistic regression tests adjusting the potential difference of age and gender for each sample. The calculation of odds ratio (OR) is also based on the risk allele of each SNP.

Inconsistent with the report by Berta et al. [14], we failed to replicate the association of SNP rs17158558 with HSCR. Among those replicated variants, SNP rs2435357 demonstrated the most significant association. Three of the fifteen SNPs are located in the exonic region, and the remaining twelve SNPs are located in the intronic and intergenic region respectively. RegulomDB annotations [15], a database which provides functional annotations of SNPs in the human genome were investigated to identify SNPs in or near regulatory elements. Three SNPs including rs2506030, rs2435357 and rs2742234 showed high annotation score reflecting highly possibility to affect binding and link to the expression of RET (Supplementary Table 1). Except of rs2435357, the regulatory mechanism of rs2506030 and rs2742234 contributing to disease pathogenesis are still await for further validation.

### Independence testing of *RET* SNPs and heritability explained for HSCR

For the purpose of identifying independent variants in *RET* associated with HSCR, the LD patterns (r^2^) of the fifteen SNPs are examined based on our replication results and public data including in Asians and Caucasians (Supplementary Figure 1). The LD patterns in East Asian populations and our study were similar. In Caucasians, SNPs showed less comprehensive LD structures comparing with our study showing limited pairwise SNPs correlation (r^2^ < 0.2). To figure out the Chinese substructures for disease association, ten SNPs with pairwise r^2^ <0.7 were kept for further independence test (Supplementary Figure 2). Pairwise independence tests of the ten SNPs were performed by logistic regression. As shown in Table 2a, two SNPs including rs2506030 and rs2434357 remains significance after adjusting for the effect of other individual SNPs. SNP rs2434357 exhibited the strongest contribution reflected by the most significant P value after adjustment. This finding was consistent with previous studies in Caucasians, showing a potential regulatory role for this SNP to HSCR. SNP rs2506030 (chr10: 42952399) was also identified as an independent contributor, which is consistent with the long physical distance to the big LD block in *RET* centered by rs2435357 (chr10: 43086608). We also observed the effect of potential independent SNPs was mainly covered by the effect of SNP rs2435357. Controlling the effect of rs2506030, all the tested SNPs remain significant (Table 2a).

**Table 2a:** Independence test by adjusting for the effects of other SNPs in the RET region

SNP	SNP whose effect was adjusted for *									
	rs2506030	rs2506011	rs2435357	rs2505532	rs2565206	rs1800860	rs2742234	rs2742236	rs7893332	rs2505526
**rs2506030**	NA	2.1E-13	4.4E-02	2.1E-05	4.1E-06	1.6E-07	7.0E-04	1.3E-08	4.3E-09	5.9E-06
rs2506011	5.0E-39	NA	4.9E-01	3.2E-02	1.9E-40	7.7E-30	2.3E-04	6.0E-17	1.1E-30	2.2E-14
**rs2435357**	3.4E-69	5.0E-41	NA	4.7E-31	3.0E-64	3.2E-67	2.5E-21	4.7E-51	2.0E-68	1.2E-46
rs2505532	1.3E-41	1.3E-12	7.1E-01	NA	1.6E-53	1.3E-40	2.9E-05	1.6E-27	1.2E-39	4.7E-20
rs2565206	4.4E-10	7.7E-18	9.8E-01	4.5E-23	NA	3.6E-06	3.9E-03	4.0E-04	3.0E-13	1.0E-08
rs1800860	3.2E-07	1.3E-03	4.7E-03	2.9E-04	1.6E-02	NA	5.9E-01	1.6E-05	4.8E-05	3.1E-02
rs2742234	1.3E-47	3.8E-22	1.1E-01	6.8E-14	8.3E-45	7.6E-45	NA	2.3E-28	1.8E-48	4.5E-24
rs2742236	1.7E-27	1.2E-07	8.3E-01	7.0E-09	1.7E-20	2.3E-25	9.7E-01	NA	1.4E-33	1.4E-09
rs7893332	4.1E-07	7.6E-01	3.4E-01	5.7E-01	2.3E-07	3.7E-02	1.2E-01	7.7E-12	NA	4.7E-01
rs2505526	5.0E-28	3.4E-11	7.3E-01	3.2E-05	2.3E-28	3.6E-25	9.8E-01	1.3E-13	1.1E-26	NA

* The data in each column represent the remaining effect of association (P-values) after adjusting for the effect of SNP(s) on the top row of each column

In order to further clarify the independent candidature of the SNPs other than rs2506030 and rs2435357, stepwise logistic regression analysis was further performed to test the independence of these SNPs. As mentioned above, the effect of rs2506030 was relatively standalone, we only consider the nine contiguous SNPs from rs2506011 to rs2505526. Consistently with pairwise logistic regression results, the analysis showed that rs2435357 exhibited the strongest association with HSCR (*P*=2.5E-10). Subsequent addition of SNPs rs2505532 (*P*=0.011), rs1800860 (*P*=0.024) and rs2742234 (*P*=0.008) also significantly improved the model, providing further evidence that variants in the region are independently associated with the disease. Further addition of rs2742236 (*P*=0.055) showed marginal improvement to the model (Table 2b). These results identified the notion that diversified effects of five independent SNPs including rs2506030, rs2435357, rs2505532, rs1800860, rs2742234 exist in this region for disease susceptibility.

**Table 2b:** Significant results from stepwise logistic regression of the six SNPs in the RET region

SNPs*	*P*_value	OR (CI 0.95)
rs2435357_T	2.5E-10	3.31(2.29~4.81)
rs2505532_C	0.011	1.49(1.09~2.03)
rs1800860_G	0.024	1.33(1.04~1.71)
rs2742234_C	0.008	1.55(1.12~2.14)
rs2742236_G	0.055	1.39(0.99~1.95)

SNPs significantly improved the disease association model was shown

Haplotype analysis was also performed using logistic regression on the five SNPs: rs2506030, rs2435357, rs2505532, rs1800860, rs2742234 and the SNP rs2742236 showed marginal significance of indepen-dence to disease. The result indicated that G-T-C-G-C-G haplotype formed by the risk alleles of the six SNPs was the major risk haplotype with a combined OR of 2.37 and P-value of 5.18E-54, whereas the G-C-T-G-T-A formed the major protective haplotype with combined ORs of 0.46 and P-value of 9.35E-22. More than one haplotypes were observed as risk combinations associated with HSCR, the omnibus association for all the haplotypes were identified with a high confidence (P-value of 2.12E-75). It seems that the haplotype associations have a greater effect size than most individual SNPs alone (OR equal to 2.98 for the haplotype vs. OR equal to 2.80 for the most associated SNP rs2435357) (Table 3). Thus, it would be of value to exam whether epistatic interaction exists among the six SNPs.

**Table 3:** Association of the haplotypes derived from six independent SNPs in RET identified in current study

rs2506030	rs2435357	rs2505532	rs1800860	rs2742234	rs2742236	F_A	F_U	*P*	OR	*P*_OMNIBUS
Risk haplotype*										2.12E-75
G	T	C	G	C	G	0.56	0.35	5.18E-54	2.37(2.05~2.76)	
A	T	C	G	C	G	0.1	0.08	7.77E-03	1.29(1.00~1.67)	
Protective haplotype*										
G	C	T	G	T	A	0.1	0.19	9.35E-22	0.46(0.37~0.57)	
A	C	T	G	T	G	0.04	0.07	7.39E-10	0.46(0.33~0.65)	
G	C	T	A	T	G	0.05	0.07	5.40E-05	0.62(0.45~0.85)	
G	C	C	A	T	A	0.02	0.05	5.65E-06	0.50(0.33~0.75)	

* Haplotypes with minor haplotype frequency in controls larger than 0.05 were shown.

F_A/F_U indicates risk haplotype frequency of the SNP in cases or controls. The P value indicates the significance based on haplotypic association tests.

*P*_OMNIBUS indicates the omnibus association significance across all the risk haplotypes and protective haplotypes.

### Intragenic SNPs showing epistatic effect for HSCR

The associated SNPs may influence the disease risk individually (main effects) or behave jointly (epistatic interactions) [16]. Pairwise epistasis test using PLINK (based on logistic regression analysis) was performed. As shown in Table 4 (right top panel), the results suggested multiple significant effects from epistatic interaction among the six variants. SNPs rs2505532 and rs2742234 showed the strongest evidence of interacting effect with the disease (*P*=8.45E-15, OR=1.98). SNPs rs1800860 showed less synergetic effect with other SNPs reflected by the insignificant *P* values shaded in grey. This phenomenon may indicate the true signals or affect by the limited power through logistic regression. Another statistical method for testing epistatic inter-action without considering the main effect of association was also applied for further validation. Pairwise Multifactor dimensionality reduction (MDR) analysis was adopted here to test epistatic interaction between SNP pairs. Table 4 (left bottom panel) showed the results of cross-validation consistency (CVC) and Balanced accuracy (BA) obtained from MDR analysis of the two-locus model, which showed significant pairwise interactions. In agreement with the results from logistic regression, all the genetic interacting effect between SNPs pairs using MDR were identified, including SNP pairs rs1800860 with rs2506030, rs2435357 and rs2742234 respectively.

**Table 4:** Pair-wise epistatic interacting results among six independent variants in RET done by logistic regression and Multifactor dimensionality reduction (MDR)

		rs2506030	rs2435357	rs2505532	rs1800860	rs2742234	rs2742236
		Logistic Regression					
rs2506030	Multiple Dimension Reduction	NA	OR=1.36(1.14 ~1.63) P=7.57E-04	OR=1.41(1.17~1.69) P=2.39E-04	P=0.32	OR=1.23(1.03~1.47) P=0.03	OR=1.32(1.07~1.62) P=0.00816
rs2435357		CVC=10/10 BA=0.68 OR=5.41(4.58~6.38)	NA	OR=1.71(1.44~2.02) P=8.50E-10	P=0.2	OR=1.73(1.47~2.03) P=6.94E-11	OR=1.50(1.24~1.80) P=2.46E-05
rs2505532		CVC=10/10 BA=0.64 OR=3.48 (2.98~4.05)	CVC=10/10 BA=0.69 OR=5.25(4.46~6.18)	NA	OR=1.64(1.35~2.01) P=1.12E-06	OR=1.97(1.66~2.34) P=8.45E-15	OR=1.87(1.56~2.23) P=9.99E-12
rs1800860		CVC=10/10 BA=0.57 OR=1.76(1.51~2.04)	CVC=10/10 BA=0.69 OR=5.25(4.46~6.18)	CVC=10/10 BA=0.65 OR=3.37(2.90~3.92)	NA	P=0.16	OR=1.28(1.03~1.59) P=0.03
rs2742234		CVC=10/10 BA=0.66 OR=4.39(3.72~5.18)	CVC=10/10 BA=0.69 OR=6.21 (5.23~7.37)	CVC=10/10 BA=0.67 OR=4.14(3.54~4.84)	CVC=10/10 BA=0.66 OR=4.39(3.72~5.17)	NA	OR=1.83(1.49~2.25) P=1.13E-08
rs2742236		CVC=10/10 BA=0.61 OR=2.47(2.13~2.87)	CVC=10/10 BA=0.68 OR=5.12(4.36~6.03)	CVC=10/10 BA=0.65 OR=3.90(3.32~4.58)	CVC=10/10 BA=0.61 OR=2.49(2.15~2.89)	CVC=10/10 BA=0.67 OR=4.70(3.98~5.55)	NA

OR means odds ratio for interaction, and a value of 1.0 indicates no effect. Cross-validation consistency (CVC) reflects the number of times MDR analysis identified the same model as the data were divided into different segments. Balanced accuracy is defined as (sensitivity + specificity)/2.

### Clinical stratification of multiple SNPs in *RET* with HSCR

Since HSCR is an extremely heterogeneous disease, we went on to ask the question whether the risk alleles of independent contributors in *RET* are also associated with different manifestations in our study. Regarding to the length of the affected segment, HSCR patients was classified into S-HSCR, L-HSCR and TCA. The subclinical information was collected as shown in Supplementary Table 2. All six SNPs were observed to have consistent patterns as associated with different

types of aganglionsis status through subphenotype-control analysis, including S-HSCR versus controls, L-HSCR versus controls and TCA versus controls respectively (Table 5). The OR of S-HSCR, L-HSCR and TCA to controls for all six SNPs were plotted, we observed the effect size is inverse proportional to the length of the affected segment (Supplementary Figure 3). It seems the common independent associated variants are more likely to affect the S-HSCR patients rather than L-HSCR and TCA patients. Case-only analysis was also conducted across different manifestations to further demonstrate the observations. Linear regression was adopted to compare the genetic effect size to the disease status. As presented in Table 5, we observed that the six SNPs showed significant *P* values (4.2E-05≤ *P*_quantitative ≤ 6.9E-02) reflect-ing the genetic difference depending on the extent of the aganglionic segment of HSCR. However, we failed to replicate the genetic predisposition in gender manner upon the replicated six SNPs in *RET* (Supplementary Table 3), which was mentioned in previous study [17].

**Table 5:** The association results of six independent SNPs in RET to different subclinical features classified by aganglionosis length including short-length(S-HSCR), long-length (L-HSCR) and TCA

SNP		*P*_Quantitative	S-HSCR			L-HSCR			TCA		
			1033 cases			294 cases			82 cases		
	A1/A2		F_A	*P*	OR	F_A	*P*	OR	F_A	*P*	OR
rs2506030	G/A	6.40E-03	0.81	1.70E-08	1.52	0.8	1.40E-02	1.33	0.71	0.42	0.86
rs2435357	T/C	4.20E-05	0.74	5.10E-64	3.16	0.68	7.00E-19	2.55	0.59	4.10E-04	1.87
rs2505532	C/T	5.30E-03	0.77	8.10E-37	2.38	0.72	1.20E-10	2.01	0.68	5.70E-03	1.67
rs1800860	G/A	6.90E-02	0.85	9.70E-09	1.59	0.83	5.10E-03	1.43	0.8	0.7	1.09
rs2742234	C/T	2.40E-03	0.71	2.60E-45	2.58	0.66	5.10E-12	2.03	0.62	1.40E-03	1.76
rs2742236	G/A	1.40E-03	0.83	6.70E-27	2.27	0.77	8.50E-05	1.56	0.77	1.80E-02	1.62

*P*_Quantitative: The patient-only linear regression test among three subclinical groups including S-HSCR, L-HSCR and TCA; F_A indicates risk allele frequency of the SNP in each subclinical group.

## DISCUSSION

HSCR has been identified as a complex disease with strong genetic component. *RET* is the first identified susceptibility gene as associated with HSCR. Though serial polymorphisms in RET have also been illustrated to have associations with HSCR, the independent contributions of these sporadically reported SNPs were still unclear. In current study, we focused on 16 SNPs centered by SNP rs2435357 in *RET* through a total number of 1470 cases and 1473 controls matched geographically and ethnically. Six of them were iden-tified as independent contributors to HSCR with a total explained heritability equal to 9.90% (1.6E-65≤ *P*_adj≤ 2.3E-08). The synergistic effect among the six SNPs was firstly highlighted through logistic regression and MDR. We also found genetic susceptibility at these SNPs was inverse proportional to the segment lengths of the HSCR patients.

Independent contributors were often overlooked in GWAS study and subsequent replication steps, and the validation of their effects may require much larger sample sizes than are available in most studies. Using the largest replication HSCR cohort reported so far, independent effects found from the six variants in *RET* were supported by cross validation of pairwise logistic regression and stepwise regression analysis. Indepen-dent associated SNP rs2435357, rs2742234 and rs2506030 were annotated as regulatory factors using RegulomeDB. Haplotype-based association was also examined reflecting higher risk for haplotypes than the association of each individual SNPs in terms of effect size. These results provided evidence that “the variance explained by the multiple effects of independent contributors in a locus was much higher than the variance explained by the single reported SNP effect”. There exists certain possibility that currently identified susceptibility variants for HSCR in *RET* are just the tag-SNPs that have high LD with an unknown functional variant. Therefore, it is possible that even more variants may be independently associated with HSCR in this region of high interest and identification of their roles requires further investigation in the future.

We tested pairwise interaction among the SNPs by logistic regression showing strong synergistic effect among the six identified independent SNPs in *RET*. However, logistic regression based methods are often criticized for their inability to deal with non-linear models and with high dimensional data that contain many potentially interacting predictor variables. It could be argued that the interaction effect detected in the present study might simply reflect a haplotype effect which implicates a single risk variant effect for HSCR. MDR seeks to identify evidence for higher-order genetic interactions in the absence of statistically significant main effects to the disease. We observed MDR analysis in the present study revealed a two-locus model among the six independent contributors. The combinations of low- and high-risk groups were classified in this model. The high-risk genotype combination for each SNP pair was consistent with the single SNP risk genotype. We also observed that the higher risk combination conferred 2.49 to 6.21 thres-holds effect size (interacting pair rs2742236 and rs1800860, rs2742234 and rs2435357) for developing HSCR. Clearly in such a manner, it may bring us into a new perspective about gene network construction. Based on this study, we can make a relationship between SNPs and genes in systems biology perspective.

In our study, we focused on the evaluation of the possible influence of the variants on aggressiveness of the disease and their role in gender manifestation differences. Interestingly, the risk of variant alleles was highly elevated in S-HSCR patients compare to L-HSCR patients and TCA patients. This result demonstrated the variants in *RET* identified in current study mainly explained the sporadic cases with short segment aganglionosis to some extent. It would be natural to raise a question that whether there exist common associated variants may affect severe cases such as long-segment aganglionosis and total colon aganglionsis respectively. Our recent study identified a novel HSCR susceptibility gene, which is complemen-tary to the common variants in *RET* identified in study, significantly elevated risk in severe HSCR cases (manuscript in preparation). It should be noted that the significance is before correction for multiple testing and the sample size among S-HSCR, L-HSCR and TCA patients was unbalanced, which may lead to false positive discovery and false negative results. To conquer this, a definitive conclusion for subclinical association requires a significant increase in sample size and/or an independent replication work in the same cohort. Elucidating connections between genetic variations and clinical manifestations, especially the life-threatening L-HSCR and TCA ones, would substantially help to clarify the disease mechanisms and further to improve clinical intervention.

Emison et al. [17] mentioned the different roles of common and rare variants in *RET*, suggest that the *RET* genetic effect in the severe cases is more likely cause of the congregation of common variants in patients with rare coding mutations. Hence, to further elucidate the key role of *RET* to HSCR, including different subclinical status, the association of common variants should not be neglected. Integrating with the rare mutation through unknown mechanism, we may go one step further to better understand the mechanism of the disease. They also observed that common variants in *RET* shows different genetic effects in males and females using 126 probands and their parents. However, in our cohort, no gender effect was observed between males and females. The inconsistency can be explained by the potential population difference, for which the previous findings were based on Caucasian populations as we mentioned in our results the LD structures between Asians and Europeans are discrepant (Supplementary Figure 1). Of course, further study is needed to explore the potential population difference according to the disease etiology.

In summary, although the detailed mechanisms still remain largely undetermined, we identified six common variants as independently associated with HSCR status, there was significant synergistic epistatic interaction across the six risk variants in *RET*, Our study proposed a link that may help bridge the gap between genetic susceptibility and subclinical manifestation, which is an important next step for association studies in order to better understand disease mechanisms and identify new drug targets. An alternative explanation is that the haplotype formed by the risk alleles of the six SNPs tags an unknown functional variant that is in high LD and is associated with the disease. To address these possibilities, further functional characterization and sequencing of the region are required in future studies.

## MATERIALS AND METHODS

### Study subjects

The samples included in the current study were collected from Guangzhou Women and Children's medical center. All the cases have diagnosed with HSCR by barium enema and anorectal manometry evaluation before surgical procedures and histological examination of biopsy specimen for the absence of the enteric ganglia and after surgery. The study was approved by the institutional review board of the hospital. The written informed consents were provided by guardians of all patient subjects. A total of 1470 sporadic patients recruited from 2000 to 2015 were all claimed as south Chinese and divided into three subgroups according to the segment lengths of aganglionosis including 1033 short segment (S-HSCR, 294 long segment (L-HSCR) and 82 total colonic aganglionosis (TCA), subsequently. The blood samples of 1473 controls matched geographically and ethnically were collected with no history of HSCR and neurological related disorders.

### SNP genotyping and quality control

Together with 74 SNPs involved in other independent study (unpublished data), 16 SNPs in *RET* were included to be genotyped by MassARRAY iPLEX Gold system (Sequenom) on all the samples. The SNP included in this study were selected accordingto the associated studies searched by NCBI following the three criteria. (1. SNP with high probability to be regulatory variants were kept for further pursuing (Regulome DB score higher than 2f. http://regulomedb.org/). (2. Removed one of the two SNPs with Linkage disequilibrium(r^2^) larger than 0.8 were kept one. (3. SNP with minor allele frequency larger than 5% in Chinese population (CHB) were kept (https://www.ncbi.nlm.nih.gov/snp/?term=). We carried out quality control steps as follows: 1. SNPs with >10% missing data were removed (1 SNPs), subjects with >5% missing data were removed. Three SNPs were removed according the genotyping allele intensity plots for clustering quality and violation of Hardy–Weinberg equilibrium (HWE). SNPs were removed if HWE P < 1.0E-04 calculated by control subjects. After quality control, all 16 SNPs were kept for further analysis consisted of 1469 cases and 1466 controls.

### Association analysis and subphenotype analysis

The SNPs were analyzed for associations with the disease by means of comparison of the minor allele frequency in patients and controls (basic allelic test) as well as other tests using PLINK1.9 (genotype test of 3 × 2 contingency tables, Cochran–Armitage trend test, test of dominant and recessive models) [18]. Association of the SNPs with disease risk was also corrected by logistic regression using age and sex as covariates and the associations found in this study remain significant. Association with subphenotype was analyzed by comparing cases with a certain subphenotype with controls, cases without the subphenotype with controls.

### Independence testing

Linkage disequilibrium patterns and values were obtained using HaploView. SNPTEST v2.5b was used to perform the logistic regression tests in this study[19]. Tests of independent contributions toward disease associations for SNPs in a single locus were done using logistic regression, adjusting for the effect of a specific SNP (COVsnp) in the same locus. Stepwise logistic regression was performed by SPSS 16.0. Briefly, variables were added to the logistic regression equation one at a time, using the statistical criterion of reducing the 2Log10 Likelihood error for the included variables. After each variable was entered, each of the included variables was tested to see whether the model would be better off if the variable were excluded.

### Haplotype analysis

First, all founders are phased using the E-M algorithm implanted in PLINK, which will generate haplotype-specific tests (1df) for both disease and quantitative traits; an omnibus association statistic will also be computed (*P*_omnibus). In all cases, the tests are based on the expected number of haplotypes each individual has. Then the association was performed on all the most likely haplotype assignments as SNPs and use all the standard analytic options (*P*). The case/control omnibus test is a H-1 degree of freedom test, if there are H haplotypes.

### Genetic epistasis

Epistasis test (case-control analysis) by logistic regression was adopted here for parametric analysis of genetic interaction using PLINK1.9 [18]. PLINK uses a model according to allele dosage ranging from 0 to 2 indicating the number of risk alleles for each SNP, A and B, and fits the model in the form of Y =b_0_ + b_1_ SNPA + b_2_ SNPB + b_3_ SNPA*SNPB + e. The parameters b1, b2 and b3 indicate the contribution of SNP A and SNP B and interaction between A and B. The test for interaction is based on the coefficient b3. P value of <0.05 was considered statistically significant.

Pairwise non-parametric epistasis test was also applied using multifactor dimensionality reduction (MDR) analysis [20]. This method includes a combined cross-validation (CV)/permutation testing procedure that minimizes false positive results by multiple examinations of the data. The statistical significance was determined by comparing the average prediction error from the observed data with the distribution of average prediction errors under the null hypothesis. The MDR analysis was carried out using version 2.0 of the open-source MDR software package that is freely available online (http://www.epistasis.org).

## SUPPLEMENTARY MATERIAL TABLES AND FIGURES


