# Supplementary material for: Large-scale replication study identified multiple independent SNPs in RET synergistically associated with Hirschsprung disease in Southern Chinese population
Source: Aging (Albany NY). 2017 Sep 20;9(9):1996–2009. doi: 10.18632/aging.101294 (PMC5636671; doi:10.18632/aging.101294)
Supplement: Supplementary file 1 [file aging-09-1996-s001.pdf]

SUPPLEMENTAL MATERIAL

Supplementary Table 1. Functional prediction of the sixteen recruited SNPs in RET by Regulome DB annotation.

#chromosome	coordinate	rsid	score
chr10	43612608	rs2742234	1f
chr10	43447846	rs2506030	2b
chr10	43582055	rs2435357	2b
chr10	43572510	rs10900297	4
chr10	43574935	rs2506011	4
chr10	43606686	rs1800860	4
chr10	43583149	rs2435356	5
chr10	43594544	rs2505532	5
chr10	43595967	rs1800858	5
chr10	43613842	rs1800861	5
chr10	43620334	rs17158558	5
chr10	43620550	rs2742236	5
chr10	43652759	rs7893332	5
chr10	43702142	rs1254958	5
chr10	43769890	rs2505526	5
chr10	43595780	rs2565206	7

Score	Supporting data
1a	eQTL + TF binding + matched TF motif + matched DNase Footprint + DNase peak
1b	eQTL + TF binding + any motif + DNase Footprint + DNase peak
1c	eQTL + TF binding + matched TF motif + DNase peak
1d	eQTL + TF binding + any motif + DNase peak
1e	eQTL + TF binding + matched TF motif
1f	eQTL + TF binding / DNase peak
2a	TF binding + matched TF motif + matched DNase Footprint + DNase peak
2b	TF binding + any motif + DNase Footprint + DNase peak
2c	TF binding + matched TF motif + DNase peak
3a	TF binding + any motif + DNase peak
3b	TF binding + matched TF motif
4	TF binding + DNase peak
5	TF binding or DNase peak
6	other

Web sources used in this study

RegulomeDB: http://regulome.stanford.edu/

Supplementary Table 2. The subclinical information collected for the subjects in this study.

HCSR subphenotype	Cases (n=1470)	%	Controls (n=1473)	%
Subjects				
Age range (Months)	8.37±20.50		18.61±19.75	
≤2	725	49.32%	458	31.09%
>2	745	50.68%	1015	68.91%
Gender				
Females	240	16.33%	967	65.65%
Males	1230	83.67%	506	34.35%
Clinical manifestation				
SHCSR	1033	70.27%		
LHCSR	294	20.00%		
TCA	82	5.58%		
Total intestine	3	0.20%		
Enteritis_before_operation	261	17.76%		
Enteritis_after_operation	249	16.94%		

Supplementary Table 3. The case-only association comparing the gender heterogeneity between females and males.

CHR	SNP	BP	A1/A2	Freq_female	Freq_male	*P*	OR	0.95 CI
10	rs2506030	42952399	G/A	0.80	0.80	0.97	1.01	(0.79~1.28)
10	rs2435357	43086608	T/C	0.71	0.71	0.90	0.99	(0.79~1.23)
10	rs2505532	43099097	C/T	0.76	0.75	0.47	1.09	(0.86~1.38)
10	rs1800860	43111239	G/A	0.84	0.84	0.96	0.99	(0.76~1.30)
10	rs2742234	43117161	C/T	0.67	0.69	0.28	0.89	(0.72~1.10)
10	rs2742236	43125103	G/A	0.80	0.81	0.38	0.90	(0.70~1.15)

freq：frequency

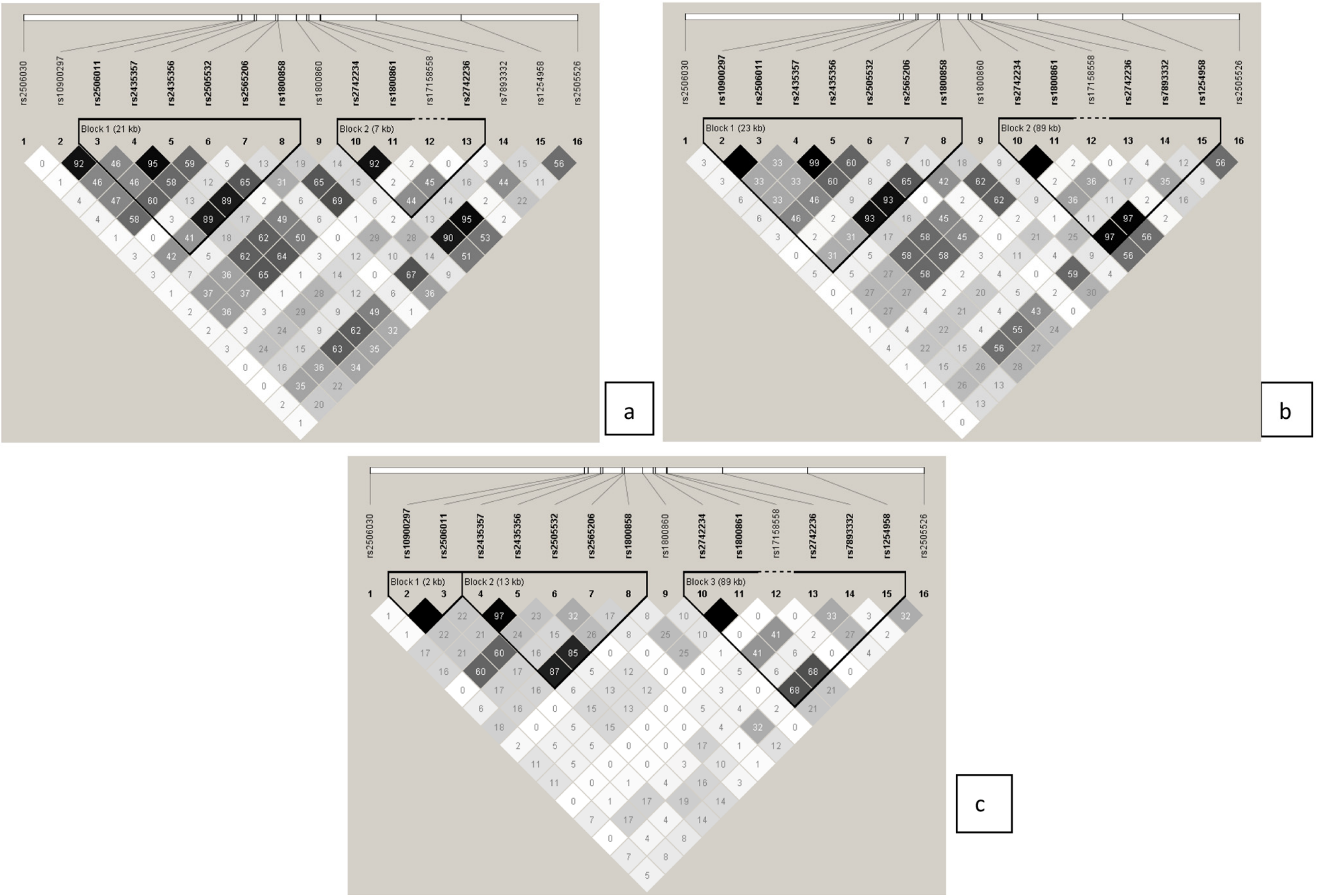

Supplementary Figure 1. The LD (r2) patterns of sixteen recruited SNPs in RET across South Chinese population in this study (**a**), Eastern Asian populations (**b**) and CEU populations (**c**) from 1000G data. The LD pattern looks less comprehensive in CEU comparing with Chinese, shows the genetic heterogeneity between the populations and may partially explain the etiology difference

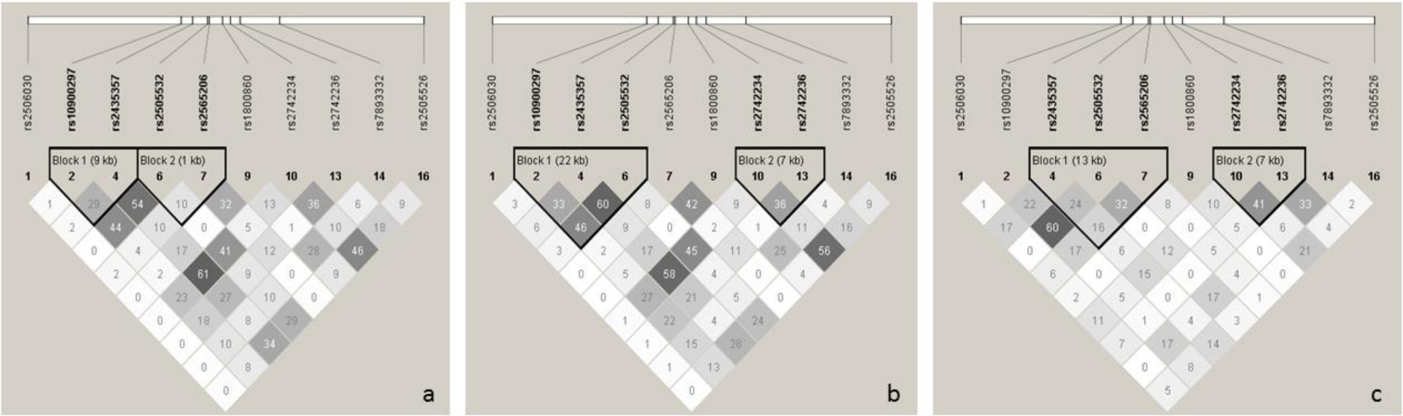

Supplementary Figure 2. The LD patterns among ten SNPs showed significant disease association in different populations. Shown are r2 values for individuals in South Chinese populations replicated in this study (**a**)，East Asian populations (CHB, CHX and JPT) (**b**)，Utah residents with ancestry from northern and western Europe (CEU) (c); The LD patterns between (a) and (b) are similar, which are slightly different with the LD pattern of (**c**)， reflecting the potential population difference.

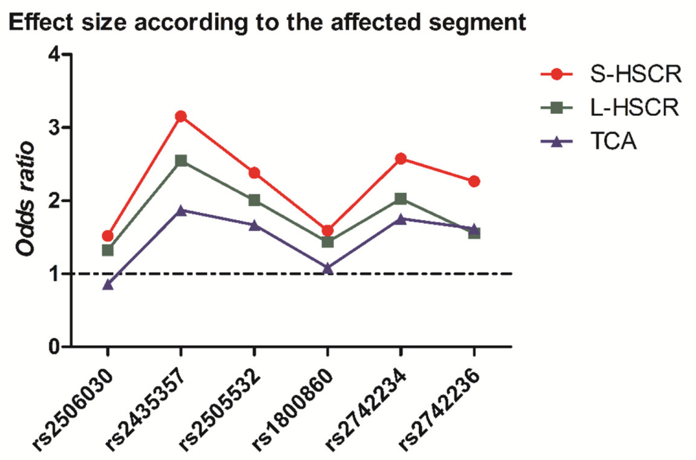

Supplementary Figure 3. The effect size (OR) comparison among three subclinical groups including short-length(S-HSCR), long-length (L-HSCR) and TCA.
